# Multiparametric liver MRI for predicting early recurrence of hepatocellular carcinoma after microwave ablation

**DOI:** 10.1186/s40644-022-00471-5

**Published:** 2022-08-30

**Authors:** Zhaohe Zhang, Jie Yu, Sisi Liu, Linan Dong, Tiefang Liu, Haiyi Wang, Zhiyu Han, Xiaojing Zhang, Ping Liang

**Affiliations:** 1 Department of Interventional Ultrasound, PLA Medical College & Fifth Medical Center of Chinese PLA General Hospital, Haidian District, No. 28, Fuxing Road, Beijing, 100853 People’s Republic of China; 2 Department of Medical Imaging, PLA Medical College & First Medical Center Chinese PLA General Hospital, Beijing, People’s Republic of China

**Keywords:** Hepatocellular carcinoma, Minimal ablative margin, Multiparametric liver MRI, Microwave ablation, Early recurrence

## Abstract

**Background:**

High early recurrence (ER) of hepatocellular carcinoma (HCC) after microwave ablation (MWA) represents a sign of aggressive behavior and severely worsens prognosis. The aim of this study was to estimate the outcome of HCC following MWA and develop a response algorithmic strategy based on multiparametric MRI and clinical variables.

**Methods:**

In this retrospective study, we reviewed the records of 339 patients (mean age, 62 ± 12 years; 106 men) treated with percutaneous MWA for HCC between January 2014 and December 2017 that were evaluated by multiparametric MRI. These patients were randomly split into a development and an internal validation group (3:1). Logistic regression analysis was used to screen imaging features. Multivariate Cox regression analysis was then performed to determine predictors of ER (within 2 years) of MWA. The response algorithmic strategy to predict ER was developed and validated using these data sets. ER rates were also evaluated by Kaplan–Meier analysis.

**Results:**

Based on logistic regression analyses, we established an image response algorithm integrating ill-defined margins, lack of capsule enhancement, pre-ablative ADC, ΔADC, and EADC to calculate recurrence scores and define the risk of ER. In a multivariate Cox regression model, the independent risk factors of ER (*p* < 0.05) were minimal ablative margin (MAM) (HR 0.57; 95% CI 0.35 – 0.95; *p* < 0.001), the recurrence score (HR: 9.25; 95% CI 4.25 – 16.56; *p* = 0.021), and tumor size (HR 6.21; 95% CI 1.25 – 10.82; *p* = 0.014). Combining MAM and tumor size, the recurrence score calculated by the response algorithmic strategy provided predictive accuracy of 93.5%, with sensitivity of 92.3% and specificity of 83.1%. Kaplan–Meier estimates of the rates of ER in the low-risk and high-risk groups were 6.8% (95% CI 4.0 – 9.6) and 30.5% (95% CI 23.6 – 37.4), respectively.

**Conclusion:**

A response algorithmic strategy based on multiparametric MRI and clinical variables was useful for predicting the ER of HCC after MWA.

**Supplementary Information:**

The online version contains supplementary material available at 10.1186/s40644-022-00471-5.

## Background

Hepatocellular carcinoma (HCC) represents the major form of primary liver cancers, and is the third most common cause of cancer death globally [1–3]. Percutaneous microwave ablation (MWA) has been widely used for treating hepatic malignancy given that it can achieve an overall survival comparable to that after surgery in patients with early-stage HCC [4]. Frequent recurrence is associated with worse survival in HCC after MWA [5]. Despite potential curative efficiency and surveillance in MWA, early recurrence (ER) after ablation remains a major challenge. Indeed, up to 70% of patients with HCC combined with cirrhosis experience recurrence after MWA, especially during the first two years after ablation, which challenges accurate prognosis [6, 7].

Although MWA is a curative treatment for HCC, it precludes the possibility to conduct histopathological examination of the whole tumor. In particular, patients marked as treatment response equivocal in post-ablation assessment can only be assessed through the absence of residual tumors at 1-month follow-up imaging or through the absence of local tumor progression (LTP) and metastases at subsequent follow-up imaging [8]. While a preoperative puncture biopsy can provide important prognostic information, this procedure is difficult to perform for tumors located in challenging locations, such as below the diaphragm or near important vessels [9]. Considering the risks and costs of biopsy, it is crucial to develop an approach to minimize invasive procedures.

Previous studies have shown that the imaging features of MRI can reflect the biological behavior of tumors and provide important supplementary information on tumor prognosis [10–13]. Some MRI findings, including intratumoral artery, ill-defined tumor margin, absence of tumor capsule, peritumoral enhancement in the arterial phase, and peritumoral hypointensity in the hepatobiliary phase, may reflect the aggressive nature of HCC [14]. The quantitative parameter apparent diffusion coefficient (ADC) has also been shown to correlate with tumor grading and microvascular invasion [12]. Thus, there may be a role for dynamic MRI, and imaging results may help to select patients with HCC who would not perform well after curative ablative therapy and who may be potential candidates for postoperative supplementary treatment strategies and clinical trials.

The purpose of this study was to investigate the correlation between image features and oncologic outcomes after MWA in HCC patients, and to suggest an imaging algorithmic strategy based on multiparametric MRI for noninvasive prediction of ER in HCC patients.

## Methods

### Study population and inclusion criteria

This retrospective study was conducted in accordance with the principles of the Declaration of Helsinki. The Ethics Committee of Chinese PLA General Hospital waived the requirement for written informed consent because of the retrospective nature of this study. Between January 2014 and December 2017, 1032 patients underwent MWA for HCC at the Department of Interventional Ultrasound at Chinese PLA General Hospital. Among them, 339 patients meeting the Milan criteria and undergoing MWA as a first-line treatment were included for study. A diagnosis of HCC was established in accordance with the guideline of the European Association for the Study of the Liver (EASL) or by histological review. The inclusion criteria were as follows: (A) histologically or radiologically confirmed HCC; (B) Child–Pugh A or B cirrhosis (Eastern Cooperative Oncology Group, ECOG 0); (C) single tumor with a maximum tumor size of 5 cm or less, or two to three tumors with a tumor size of 3 cm or less; (D) no evidence of vascular invasion or extrahepatic metastasis; and (E) evaluated by MRI including DWI before and after MWA. The exclusion criteria were as follows: (A) other local regional therapies or systemic treatments before MWA; (B) suboptimal image quality of MRIs; and (C) patients with portal vein tumor thrombosis or multiple metastases.

Clinical information and laboratory data for all patients were retrospectively collected from their electronic medical records. Specifically, we recorded patient demographic and survival data; any etiology of chronic liver disease; Child–Pugh classes; and levels of alpha-fetoprotein (AFP). Albumin-bilirubin (ALBI) scores were calculated from serum albumin and bilirubin [15].

### MRI protocol

All examinations were performed using a 3.0-T MRI system (GE Signa 3.0 T HDX TWINSP, America) with a dedicated 18-channel system before and after MWA. The detailed MRI acquisition protocol is provided in the Supplementary material.

### MWA procedures

MWA was performed under general anesthesia using ultrasonographic guidance (GE LOGIQ E9, USA) by a panel of three interventional radiologists with more than five years of experience in percutaneous MWA. MWA was carried out using a microwave instrument with a water circulation cooling system (Kangyou Nanjing; output frequency, 2450 MHz, output power, 10 – 120 W and a ky-2450 ablation needle), and a microwave needle with a 0.5 or 1.0 cm effective tip of antenna tip. Before ablation, an 18G biopsy needle was used to puncture the lesion to obtain two to three tissue biopsies for pathological examination. After completion of each biopsy, the ablation needle was used to sequentially puncture the preoperative planned site under ultrasonic guidance. Each ablation procedure was performed for 3 – 5 min, and we often used multiple overlapping ablation techniques to create a larger ablation area until the tumor was completely destroyed with the goal of achieving an ablative margin of 5 mm.

### Histopathological evaluation

In preoperative puncture biopsies, histopathological features of each tumor, including the histological grade and Ki-67 expression levels, were assessed by a liver pathologist. Histological grades were classified as well-differentiated, moderately differentiated, or poorly differentiated according to the Edmonson–Steiner nuclear grading system. When different histological grades were present in a tumor at the same time, the major grade was used as the tumor grade. Positive Ki-67 staining was defined as the presence of brownish-yellow granules in the nucleus. Tumor cells that did not stain or tumor with < 10% of tumor cells staining negative (-), and those with ≥ 10% of tumor cells staining positive ( +) were used to calculate the Ki-67 labeling index. The Ki-67 labeling index was calculated in 10 random high-magnification fields, and 1000 tumor cells were counted. Patients were divided into a Ki-67 low-expression group (Ki-67 < 10%) and a Ki-67 high-expression group (Ki-67 ≥ 10%) [16].

### Definitions

To identify variables that may predict ER of HCC after percutaneous MWA, the following prognostic factors were evaluated: tumor location (sub-capsular and intraparenchymal as opposed to perivascular and non-vascular) and minimal ablative margin (MAM). Sub-capsular HCC was defined as a tumor less than 5 mm from the peritoneum, and intraparenchymal HCC was defined as an intraparenchymal tumor at least 5 mm from the liver peritoneum [14]. Perivascular tumors were defined as index tumors characterized by contact with the primary or secondary branches of the portal vein or hepatic vein with a diameter greater than or equal to 3 mm.

Referring to the terminology reported by Ahmed et al. [17], technical validity was defined as complete coverage of the ablation zone over the index tumor and confirmation of complete tumor ablation at 1-month post-ablative imaging follow-up. LTP was defined as the occurrence of new peripheral or nodular enhancement within 1 cm of the treated tumor, or an enlargement of the initial ablation zone. Early LTP was defined as the occurrence of LTP within 2 years of MWA. Early recurrence (ER) was defined as the presence of new intra- and/or extrahepatic lesions within 2 years of MWA. ER included LTP, intrahepatic metastasis (IDM), and extrahepatic recurrence.

### Follow-up care after MWA

MRI was used to assess treatment response three days after the last course of MWA. Radiological responses were defined using the LI-RADS treatment response categorization (LR-TR) (TR nonviable, TR equivocal, and TR viable). An additional session of MWA was performed when asymmetrical peripheral enhancement, including a dispersed, nodular, or unusual pattern, was present (LR-TR viable). If a thorough ablation was accomplished, then routine MRI and serum tumor markers were evaluated one and three months after MWA, and then at six-month intervals. Each enrolled patient was followed up for at least two years after treatment. All new tumors in the ablated lesion or at other liver sites that emerged during the follow-up period were treated with MWA, if they met the requirements for MWA.

### Image analysis

A radiologist with over 10 years of experience in abdominal MRI reviewed a randomly defined training set of 20 patients to determine imaging features to be assessed. Two abdominal radiologists (with six and seven years of diagnostic abdominal MRI experience, respectively) involved in the visual analysis of all images independently evaluated quantitative and qualitative MRI features on workstations equipped with a picture archiving and communication system (PACS, Centricity 3.0; GE Healthcare, Chicago, IL, USA). Both observers analyzed index tumors consistently. These two radiologists knew this study was regarding HCC, but they were blinded to clinical, laboratory, histopathological, and follow-up findings. For each HCC lesion, the two radiologists reported invasive MRI presentations as follows: (a) intratumoral artery; (b) ill-defined margins; (c) intratumor hemorrhaging; (d) tumor parenchymal necrosis; (e) peri-arterial phase enhancements; (f) tumor envelope enhancements; and (g) homogeneity. In case of a disagreement between the readers, the final judgment was made by the chief radiologist with over 10 years of experience. Image features are described in detail in the Supplemental material. When the images were subjected to quantitative analysis, to measure each ADC and exponential apparent diffusion coefficient (EADC) value for each tumor, an elliptical region of interest with b = 0 s/mm^2^ was first drawn within the cancerous area of the DWI and subsequently replicated in the ADC and EADC maps on the same cross-section. All regions of interest (ROIs) were developed with reference to high-resolution T2-weighted images. A circular region of interest of 200 – 300 mm^2^ was also defined on the adjacent liver parenchyma, taking care to avoid blood vessels. A total of three ROIs were drawn for each lesion at each MRI examination to obtain the ADC and EADC values. Finally, the △ADC and △EADC of each tumor and the ADC and EADC ratios of each lesion on the adjacent parenchyma (Lesion-to-liver ADC/EADC ratio) were calculated. Lesion-to-liver ADC ratio was calculated as the ADC of the tumor divided by the ADC of adjacent parenchyma. Lesion-to-liver EADC ratio was calculated as the EADC of tumor divided by the EADC of adjacent parenchyma (sTable 2).

### Statistical analysis

Normally distributed data were reported as the mean ± standard deviation (SD), while the median (range) was used for non-normally distributed data. Categorical variables were expressed as the number of cases and percentiles. For intergroup comparisons of baseline characteristics, Student's *t* test or Mann–Whitney *U* test was used; for categorical variables, the chi-square test or Fisher's exact test was used to analyze interobserver agreement for each image characteristic by calculating Cohen's kappa values. A kappa statistic of 0–0.39, 0.40–0.69, and 0.70–1.00 was considered poor moderate, and good agreement, respectively. The cutoff value corresponding to the maximum Youden index was calculated using x-tiles. Univariate and multivariate stepwise logistic regression analyses were used to determine the correlation of predictors and clinical outcomes with early LTP and ER after ablation. A nomogram was created based on multivariate regression analysis, and the C-index of the corresponding nomogram was calculated. The predictive performance of significant variables and combinations of variables was also evaluated. A Cox proportional risk regression model was used for multifactorial analysis. The Kaplan–Meier method was used to estimate the ER rate. Statistical analyses were performed using R software (version 3.5.3) and MedCalc (version 20.0.3). P < 0.05 was considered statistically significant.

## Results

### Patient profiles and characteristics

**Fig. 1:**
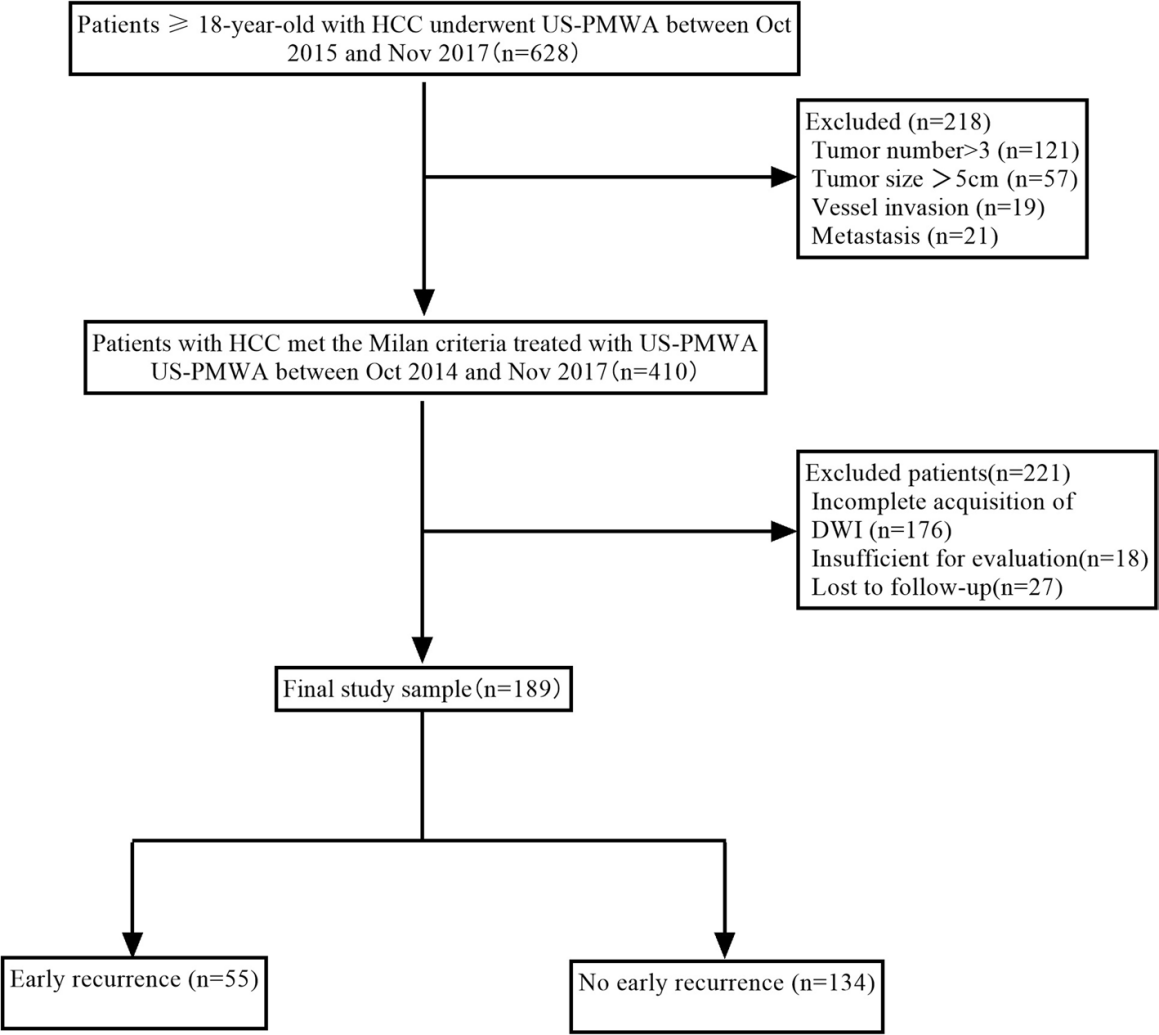
Flowchart of the inclusion and exclusion decision tree

Baseline characteristics are summarized in Table 1. Among the 1,516 consecutive patients studied, 1,177 patients were excluded. Thus, 339 patients (mean age, 62 ± 12 years; 106 men) were considered for the final analysis. Figure 1 displays a flowchart for describing the enrollment of patients. MWA techniques were effective in 97.3% (330/339) of the patients herein. A total of 115 (33.9%) patients meeting Milan criteria after MWA experienced ER by the end of the follow-up period (January 2021). A total of 99 (86.1%) patients had intraparenchymal recurrence (43 patients with local tumor progression (LTP) and 56 with distant intrahepatic metastasis (IDM)), while 16 (13.9%) patients had extrahepatic recurrence (six patients with pulmonary metastasis, 7 patients with extrahepatic lymph node metastasis, and 2 patients with bone metastasis). The median time to ER was 20.95 ± 5.81 months. All the parameters were randomly divided into development and validation data sets for prediction model construction and validation according to a 2:1 split (sTable 1). There were no differences in the early LTP rates and ER rates between the development and validation data sets after MWA (*P* = 0.23, *P* = 0.58, respectively). Univariate analysis of baseline clinical and pathological characteristics showed that larger tumor sizes, higher AFP levels, challenging tumor locations, smaller MAM and higher ALBI stage were more frequently observed in patients with ER (Table 2).

**Table 1:** Baseline characteristics of 339 patients who had undergone MWA for HCC

Characteristic	Patients (*n* = 339)
**Age (years)**	63.2 ± 9.5
**Sex (Male,%)**	190 (56.1)
**Tumor size (cm)**	
≤ 3 cm	226 (66.7)
> 3 cm	113 (33.3)
**Etiology (%)**	
HBV	303 (89.4)
HCV	32 (9.5)
NAFLD	4 (1.1)
**Child–Pugh**	
**A**	309 (91.2)
**B**	30 (8.8)
**ALBI stage**	
**I**	191 (56.3)
**II**	148 (43.7)
**AFP (%)**	
> 200 μg/L	190 (56.0)
≤ 200 μg/L	149 (44.0)
**Number of tumors (%)**	
1	292 (86.0)
> 1	47 (14.0)
**Tumor location (%)**	
Left lobe	88 (26)
Right lobe	251 (74)
Close to vessel	92 (27.0)
Close to organ/subcapsular	46 (13.7)
Close to the bile duct	12 (3.5)
Nonspecific	189 (55.8)
**Minimal ablative margin (%)**	
≤ 5 mm	113 (33.3)
> 5 mm	226 (66.7)
**Histological differentiation level (%)**	
Well-differentiated	89 (40.5)
Moderately differentiated	107 (48.6)
Poorly differentiated	24 (10.9)
**Tumor type (%)**	
Primary hepatocellular carcinoma	93 (27.5)
Recurrent hepatocellular carcinoma	246 (72.5)
**LR-TR category (%)**	
TR nonviable	250 (73.7)
TR equivocal	79 (23.3)
TR viable	10 (3.0)
**Follow-up time (months)**	23.21 ± 8.06
**Median time to LTP or metastasis (months)**	20.95 ± 5.81

Note.—Data represents the number of hepatocellular carcinomas; unless indicated otherwise, data is shown as the mean ± standard deviation for continuous variables, and number of patients with percentage in parentheses for categorical variables

*AFP* Alpha-fetoprotein, *LTP* Local tumor progression, *ALBI* Albumin-bilirubin, Time to LTP and metastasis: Time from after microwave ablation to local recurrence or metastasis, *MAM* Minimal ablative margin

**Table 2:** Comparison of baseline demographic, biochemical, and histopathological characteristics of HCC patients with and without ER

Characteristics	Total (*n* = 339)	Early recurrence (*n* = 115)	No early recurrence (*n* = 224)	*P* value
**Sex (**%**)**				
Women	134	42 (36.5)	92 (41.2%)	0.356
Men	195	63 (63.5%)	132 (58.5%)	
**Age (years)**		48.66 ± 9.87	53.62 ± 10.55	0.614
**Tumor size (**%**)**				0.021*
≤ 3 cm	186	84 (73.3)	102 (45.6)	
3 – 5 cm	152	31 (26.7)	121 (54.4)	
**Cause of disease (**%**)**				0.089
Chronic hepatitis B	305	103 (89.6)	202 (90.2)	
Chronic hepatitis C	28	10 (8.7)	18 (8)	
Nonalcoholic steatohepatitis	6	2 (1.7)	4 (17.8)	
**BCLC stage (**%**)**				0.105
A	309	105 (91.3)	204 (91.1)	
B	30	10 (8.7)	31 (8.9)	
**ALBI stage (**%**)**				0.020*
I	191	42 (36.5)	149 (66.5)	
II	148	73 (63.5)	75 (33.4)	
**Tumor location (**%**)**				< 0.001*
Close to vessel	100	75 (65.5)	25 (11.2)	
Close to organ/ subcapsular	50	19 (16.3)	31 (13.7)	
Nonspecific	191	21 (18.2)	168 (76.1)	
**Preoperative serum AFP level (**%**)**				0.021*
≤ 200 ng/mL	191	81 (70)	110 (49)	
> 200 ng/mL	148	34 (30)	114 (51)	
**Tumor type (**%**)**				0.681
Primary HCC	200	66 (58)	134 (60)	
Recurrence HCC	139	49 (42)	90 (40)	
**MAM (**%**)**				0.011*
≤ 5 mm	110	81 (70)	29 (13)	
> 5 mm	229	34 (30)	195 (87)	
**LR-TR category (**%**)**				0.550
TR nonviable	250	89 (77)	161 (72)	
TR equivocal	79	23 (20)	56 (25)	
TR viable	10	3 (3)	7 (3)	
**Ill-defined margins (**%**)**				< 0.001*
Yes	183	92 (80)	91 (41)	
No	156	23 (20)	133 (59)	
**Lack of capsule enhancement (**%**)**				< 0.001*
Yes	185	82 (71)	103 (46)	
No	154	33 (29)	121 (54)	
**ADC (**%**)**				< 0.001*
≤ 1.272 × 10^−3^ mm/s	184	98 (85)	86 (38)	
> 1.272 × 10^−3^ mm/s	155	17 (15)	138 (62)	
**ΔADC (**%**)**				< 0.001*
≤ 0.283 × 10^−3^ mm/s	179	96 (83)	83 (37)	
** > **0.283 × 10^−3^ mm/s	160	19 (17)	141 (63)	
**EADC**				0.012*
≤ 0.316	201	79 (69)	122 (54)	
** > **0.316	138	36 (31)	102 (46)	

Note: unless indicated otherwise, data represent the number of hepatocellular carcinomas, and data in parentheses represent percentages. * *P* < 0.05

a values are the mean ± standard deviation; b values are medians (ranges)

Categorical variables were compared by using a chi-square test or a Fisher’s exact test;

*AFP* Alpha-fetoprotein, *MAM* Minimal ablative margin, *ALBI* Albumin-bilirubin, *ADC* Apparent diffusion coefficient, *eADC* Exponential apparent diffusion coefficient

ALBI score = (log10 bilirubin × 0.66) + (albumin ×  − 0.085)

Classified into three grades (grade 1, ALBI score ≤  − 2.60; grade 2, − 2.60 < ALBI score ≤  − 1.39; grade 3, ALBI score >  − 1.39)

### Association between MRI imaging features and early recurrence

**Fig. 2:**
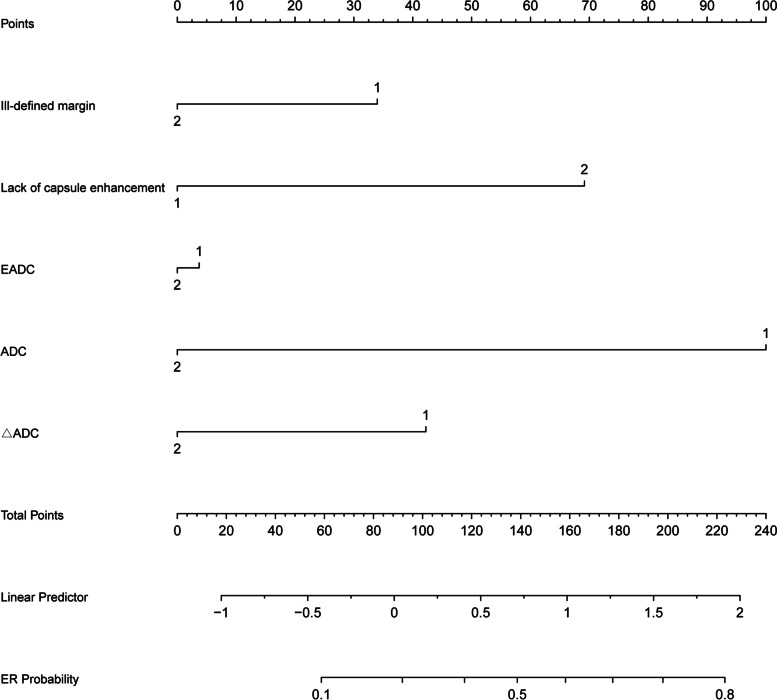
Nomogram to predict the probability of early recurrence (ER) of hepatocellular carcinoma (HCC) after microwave ablation (MWA). Predictor points are found on the uppermost point scale that corresponds to each variable. On the bottom scale, points for all variables are added and translated into the probability of HCC with progenitor phenotype. Generally, the maximum possible total points with all high-risk features present were around 240 at the bottom scale, and the ER probability of HCC was higher than 90% when the total points exceeded 224. Liner predictor = 0.811 × ill-defined margin (0: No; 1: Yes) + 1.208 × lack of capsule enhancement (0: No; 1: Yes) + 1.708 × ADC (0: ≥ 1.272 × 10^−3^ mm^2^/s; 1: < 1.272 × 10^−3^ mm^2^/s) + 1.081 × ΔADC (0: ≥ 0.283 mm^2^/s; 1: < 0.283 mm.^2^/s) + 0.113 × EADC (0: ≥ 0.316; 1: < 0.316) Cutoffs to generate the risk groups: ≤ 2.673 (Low risk); > 2.673 (high risk)

**Fig. 3:**
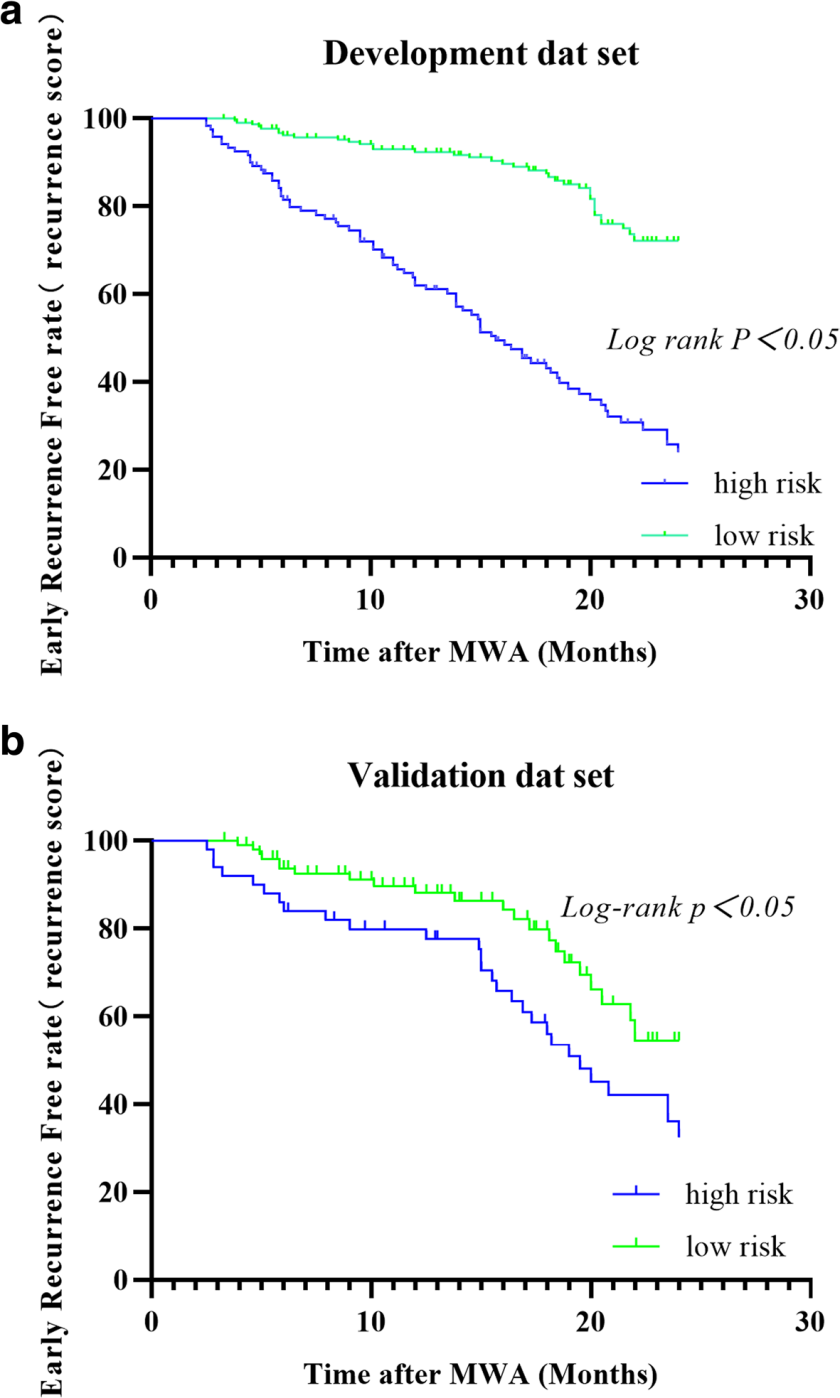
(**a**) Kaplan–Meier plot depicts early recurrence-free survival in the high-risk group and the low-risk group of development (**a**) and validation data set (**b**)

The correlation between MRI quantitative and qualitative features and Ki-67 expression was analyzed, and the features with high consistency were enrolled in the univariate and multivariate analysis. Among the MRI qualitative features (ill-defined margin and lack of capsule enhancement) and quantitative features (ADC, ΔADC, and EADC) correlated with Ki-67 expression in a way that tumors with ill-defined margins, lack of capsule enhancement, higher ADC and EADC, and lower ΔADC were more aggressive (sTable 2). The interobserver agreement for ill-defined margin (0.73), lack of capsule enhancement (0.72), ADC (0.71), ΔADC (0.71), and EADC (0.71) was higher than 0.70. The best cutoff values of ADC, ΔADC, and EADC for predicting ER were 1.272 × 10^−3^ mm^2^/s, 0.283 mm^2^/s, and 0.316 respectively, as determined by x-tile; ADC, ΔADC, and EADC were classified as binary measurements according to their respective cutoff values. The univariate and multivariate logistic regression analysis identified four parameters as potential predictors of ER, including ill-defined margins (odds ratio [OR] 2.25; 95% CI 1.31—6.12; *p* < 0.001), lack of capsule enhancement (OR 3.35; 95% CI 1.22—7.48; *p* = 0.001), ADC (OR 5.52; 95% CI 1.22—9.28; *p* = 0.001), EADC (OR 1.12; 95% CI 1.25—6.08; *p* < 0.001), and ΔADC (OR 2.95; 95% CI 1.56—7.55; *p* < 0.001) (Table 2, sTable 3). Based on the regression coefficients, an imaging prediction model was constructed and a nomogram was plotted (Fig. 2) to calculate the recurrence score for each patient. The cutoff value of recurrence score that was obtained based on the x-tile was 110 (liner predictor = 2.637), by which patients were defined as having high and low risk of ER, and the recurrence score had sensitivity of 89% and specificity of 95%. The recurrence score was associated with ER in both the development and the validation groups (*p* < 0.001) as confirmed by Kaplan–Meier survival analysis (Fig. 3). The C-index of the model was 0.851 (95% CI, 0.722—0.879) and 0.833 (95% CI, 0.715—0.863) in the validation group. sTable 3 summarizes the unadjusted and adjusted ORs for ER logistic regression.

### Predictive performance of early recurrence

As shown in Table 3 multivariate Cox regression analysis identified recurrence score and MAM to be independently associated with early LTP (HR 6.77; 95% CI 2.28—13.56; *p* = 0.035, HR 0.12; 95% CI 0.025 0.681; *p* = 0.015, respectively) and ER (HR 9.25; 95% CI 4.25—16.56; *p* = 0.021, HR 0.57; 95% CI 0.35—0.95; *p* < 0.001, respectively). Tumor location (close to vessel) (HR 7.59; 95% CI 2.35—17.58; *p* < 0.001) was a significant independent risk factor for early LTP, while tumor size was independently associated with ER (HR 6.21; 95% CI 1.25—10.82; *p* = 0.014).

**Table 3:** Multivariate cox regression analyses of variables in predicting local tumor progression and early recurrence

Predictor variables	Early LTP		*P*	ER		*P*
	Hazard Ratio	95% CI		Hazard Ratio	95% CI	
**AFP (> 200** μg/L**)**	3.87	(1.33, 6.73)	0.260	2.37	(0.85, 4.12)	0.060
**Tumor size (cm) (3 – 5 cm)**	5.78	(1.59, 9.23)	< 0.31	6.21	(1.25, 10.82)	0.014
**Tumor location (Close to vessel)**	7.59	(2.35, 17.58)	0.001	3.87	(1.35, 8.21)	0.151
**MAM (> 5 mm)**	0.12	(0.025, 0.681)	0.015	0.57	(0.35, 0.95)	< 0.001
**ALBI Stage (II stage)**	2.31	(0.95, 3.59)	0.210	1.93	(0.88, 2.87)	0.08
**Recurrence score (> 110)**	6.77	(2.28, 13.56)	0.035	9.25	(4.25, 16.56)	0.021

*Note*.—Numbers in parentheses are 95% confidence intervals (CI). *AFP* A-fetoprotein, *MAM* Minimal ablative margin, *LTP* Local tumor progression, *ER* Early recurrence, *ALBI* Albumin-bilirubin

**Fig. 4:**
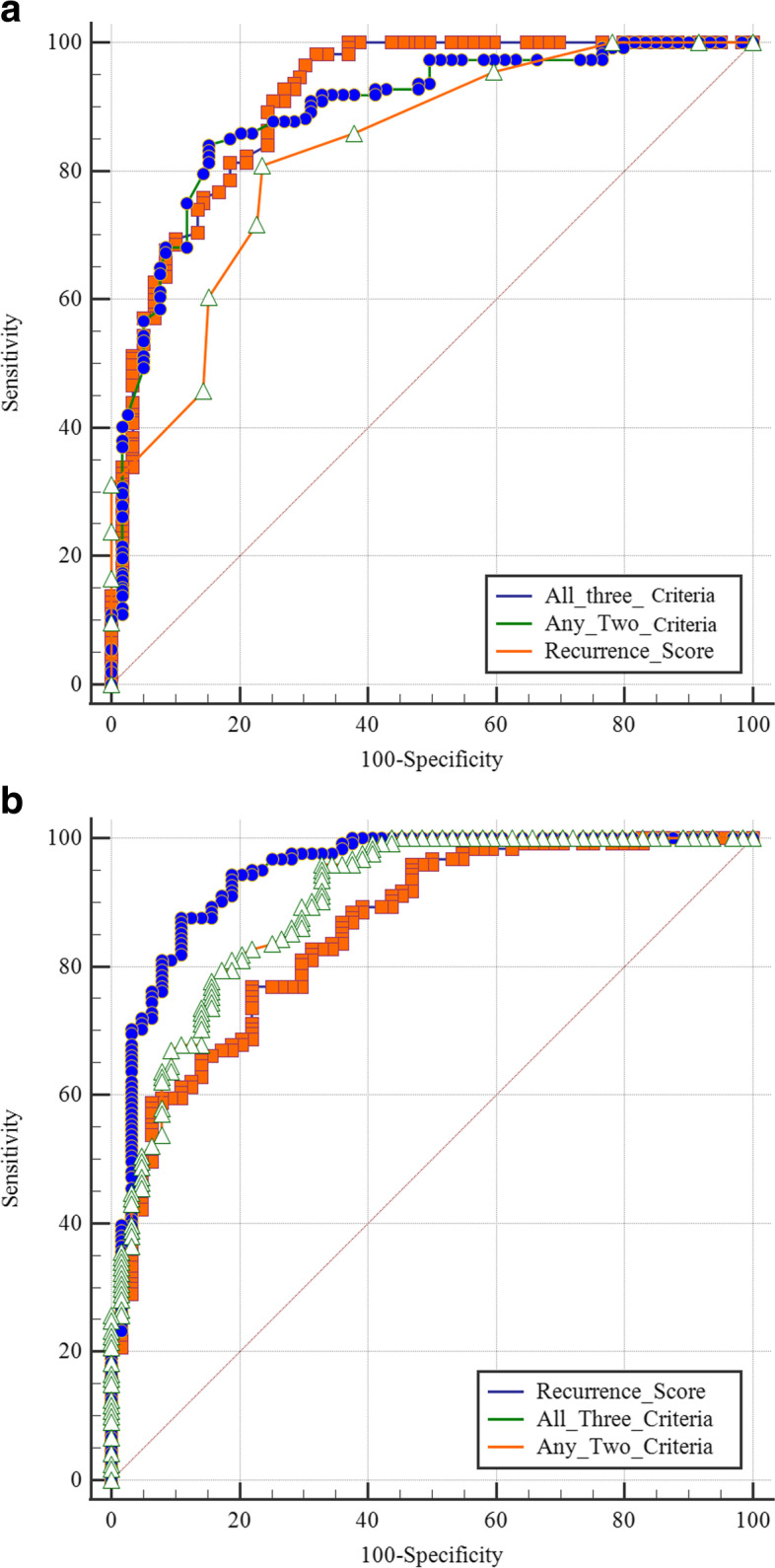
Receiver operating characteristic curves of the criteria for predicting early LTP (**a**) and early recurrence (**b**) of HCC. The criteria were the recurrence score > 110, MAM < 5 mm, and tumor size 3 – 5 cm

In predicting overall ER, a recurrence score model with a sensitivity of 71.9% (95% CI 62.9%—79.5%) and specificity of 84.1% (95% CI 77.2%—89.7%) outperformed the MAM alone. Interestingly, the recurrence score was inferior to MAM in predicting LTP. When all three criteria (recurrence score combined with MAM and tumor size) were included, specificity and sensitivity for identification of ER reached 92.3% and 83.1%, respectively (Table 4). The area under the curve (AUC) of the joint prediction model (0.849; 95% CI 0.705—0.871) had a maximum accuracy of 80.6% (Fig. 4).

**Table 4:** Predictive ability of the two identified significant criteria for the prediction of ER

Criteria	Early LTP		Early recurrence	
	**Sensitivity (%)**	**Specificity (%)**	**Sensitivity (%)**	**Specificity (%)**
**Recurrence score**				
**consensus**	63.9 (47.5, 72.6)	79.9 (73.9, 85.1)	71.9 (62.9, 79.5)	84.1 (77.2, 85.6)
**Radiologist 1**	67.5 (65.5, 82.6)	73.5 (76.2, 82.5)	75.5 (66.3, 78.2)	83.5 (84.1, 83.8)
**Radiologist 2**	60.1 (76.2, 89.4)	77.2 (78.0, 86.5)	72.1 (66.2, 76.3)	80.2 (81.5, 86.4)
**Tumor location**	60.2 (41.1, 80.2)	72.5 (56.9, 82.3)	-	-
**Tumor size**	-	-	69.2 (41.1, 78.2)	76.5 (56.5, 82.3)
**MAM**	75.7 (67.4, 88.3)	85.1 (77.2, 90.7)	60.9 (47.9 72.6)	73.1 (68.2 80.7)
**Any two criteria**	70.8 (62.5, 90.2)	87.6 (73.3, 92.5)	85.1 (78.2, 92.1)	76.9 (68.1, 83.8)
**All three criteria**	82.5 (84.0, 98.0)	88.5 (55.2, 72.8)	92.3 (86.0, 98.8)	83.1 (71.8, 90.8)

*Note*.—Numbers in parentheses are 95% confidence intervals. *AFP* A-fetoprotein, *HCC* Hepatocellular carcinoma, *MAM* Minimal ablative margin, *LTP* Local tumor progression, *ER* Early recurrence

Three criteria: Recurrence score > 110, MAM (< 5 mm), Tumor size (3–5 cm)

**Fig. 5:**
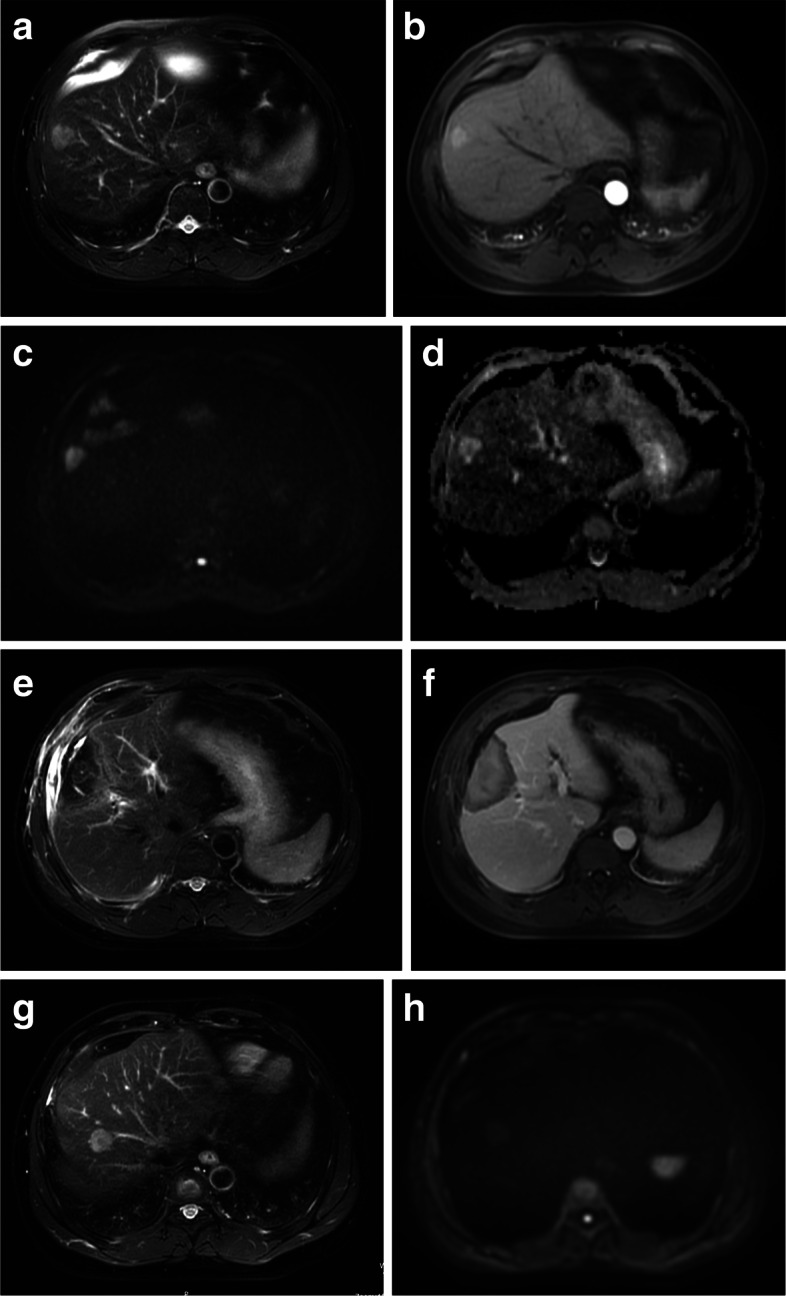
A 55-year-old man with a 2.5-cm single hepatocellular carcinoma (HCC). **A** Axial breath-triggered single-shot T2-weighted magnetic resonance imaging (MRI) showed a hyperenhancing mass in the S8 segment of the right liver near the hepatic margin. **B** Significant enhancement of the mass was seen in the arterial phase, with an ill-defined margin at the mass. **C** Axial single-shot diffusion-weighted imaging (DWI) (b = 800 s/mm^2^) and **D** apparent diffusion coefficient (ADC) maps showing a visually assessed diffusion restriction of the tumor. ADC and eADC were 0.973 × 10^−3^ mm^2^/s and 0.215, respectively, and the recurrence score exceeded the optimal cutoffs, thereby indicating a high risk of recurrence. Although a sufficient ablative margin was obtained (MAM > 5 mm), meaning that complete ablation was confirmed (**E**–**F**), tumor recurrence occurred in the right liver at 18 months after complete ablation (**G**)

### Clinical significance

**Fig. 6:**
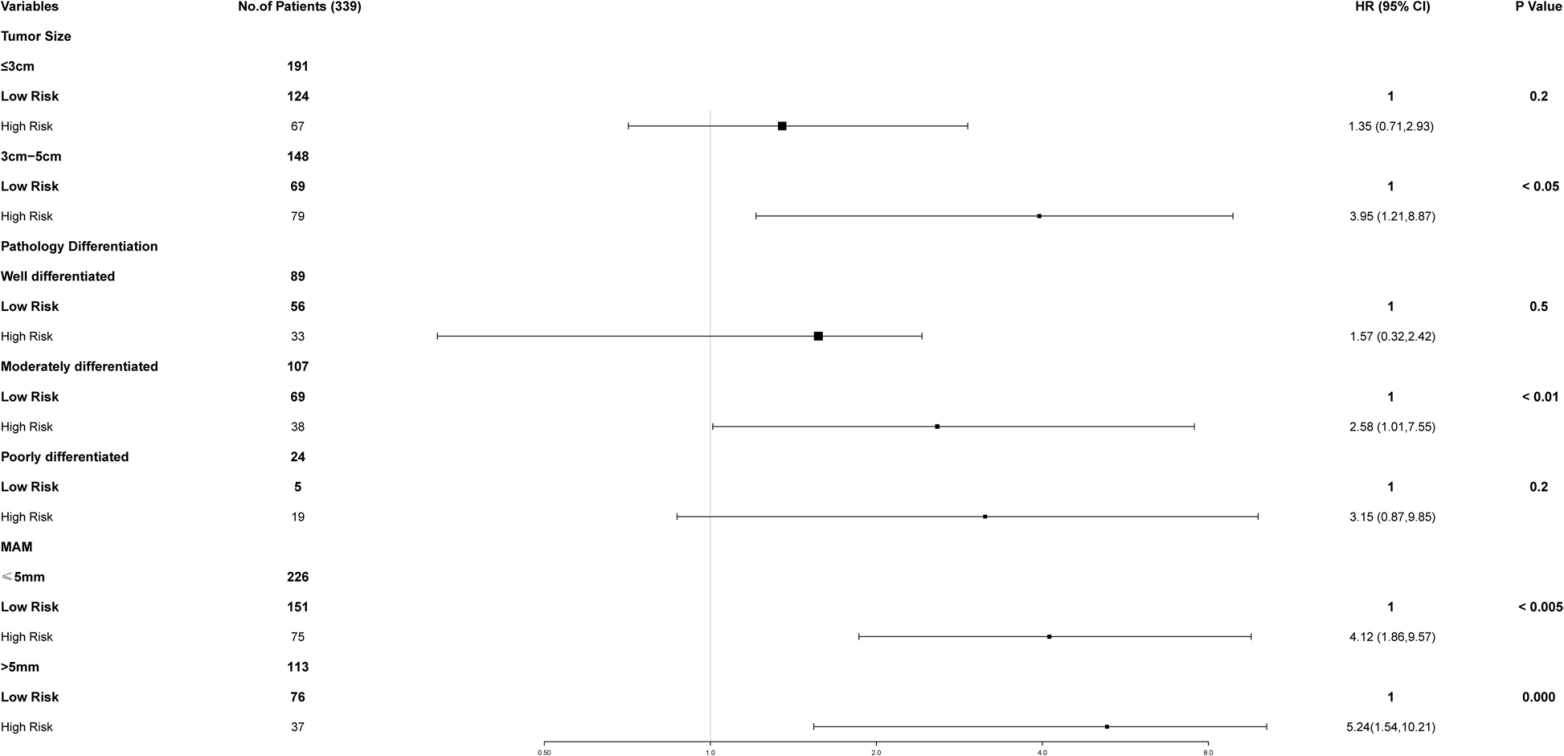
Kaplan–Meier estimates of proportion of patients free of early recurrence, according to tumor size pathology differentiation and Minimal Ablative Margin (MAM). For each group of patients, the results for low- and high-risk recurrence-score categories are shown. A low risk was defined as a recurrence score of less than 110 (liner predictor = 2.637), and a high risk was defined as a score of 110 or higher

The recurrence score predicted ER for all MAM, tumor size, and tumor differentiation subgroups (Figs. 5 and 6). The patients with a high-risk recurrence score (more than 23) had more frequent ER than patients with a low-risk score. Furthermore, not all patients that obtained favorable ablative safety margin (MAM > 5 mm) were at a low risk of recurrence. Namely, the prediction model identified 32.7% (37/113) of patients who still had a high risk of ER despite reaching adequate ablative margins (Fig. 6). More importantly, the predictive model was able to identify 35.5% (38/107) of patients with moderate differentiation levels as those having a high risk of recurrence after MWA. For larger tumors, prediction models could also identify patients at low risk of ER.

## Discussion

After MWA in HCC patients, the presence of residual tumor cells cannot be identified by conventional imaging methods [18]. Even if a favorable MAM is achieved, there is still a certain number of HCCs with ER after MWA, so follow-up is the best method to determine the ablation efficacy [19]. Our study demonstrated that tumor margins, ADC, ΔADC, and enhanced envelopes were significant predictors of HCC ER. Our regression coefficient-based nomogram indicated an individualized imaging response category to predict the risk of ER. Combined with clinical characteristics, recurrence scores provided favorable accuracy for predicting the overall ER rates. More importantly, the recurrence score could be used to quantify the risk of ER for different subtypes of MAM (≤ 5 mm and > 5 mm), tumor sizes (3–5 cm), and histopathological grades (moderately differentiated). This is useful for improving patient management, as when a high risk of ER is expected, additional adjuvant therapies might be initiated as early as possible.

MWA is a curative treatment option for early-stage HCC. To obtain adequate therapeutic response, the target tumor should be covered by the ablation zone, and a 5–10 mm margin around an index tumor is recommended [17, 20]. Lin et al. [21] have demonstrated that the ablative margin was associated with LTP and overall recurrence after radiofrequency ablation. In our study, MAM also showed favorable performance in predicting LTP, with a higher sensitivity and specificity, as confirmed in a larger sample set. However, MAM showed a limited performance in predicting overall early recurrence. Early recurrence after ablation includes LTP, intrahepatic distant metastases and extrahepatic metastases, and each of these types has a specific mechanism of pathogenesis [22]. LTP is considered related to the microscopic spread of residual tumor cells beyond the ablation margin and the local environment of a tumor (e.g., contact with blood vessels). In contrast, intrahepatic and extrahepatic metastases tend to depend on the aggressiveness and biological behavior of each tumor itself [23–25]. This fact explains the low sensitivity of MAM in predicting overall early recurrence. When the MAM was > 5 mm, the index tumor as well as most of the peritumoral infiltrative lesions were completely covered by an ablation zone, so we speculated that the factors determining early recurrence after MWA depended mainly on the aggressiveness of the tumor and the degree of tumor necrosis.

DWI is an important MRI functional imaging tool with the unique ability to display microscopic functional information such as the tissue cell structure and cell membrane integrity, and its quantitative parameters can provide information on the status of a lesion after treatment [3, 10, 12]. In our study, ADC, eADC, and changes of ADC and eADC after MWA were important imaging parameters for predicting ER after MWA. Several studies have confirmed the correlation between DWI parameters and tumor aggressiveness [26, 27]. Preoperative high levels of ADC and eADC often reflect low tumor aggressiveness and good prognosis [28, 29]. However, since thermal ablation can cause coagulative necrosis of tumor cells in a short period of time, changes in DWI parameters after MWA also reflect the degree of tumor necrosis. Most of the studies about DWI in the evaluation of the prognosis of local–regional treatment of liver cancer have focused on TACE [30, 31]. The increase in ADC after TACE is associated with increased levels of tissue necrosis and prolonged patient survival [32]. Our study demonstrated that the predictive efficacy of DWI was also applicable to ablative treatment of liver cancer. When ΔADC was greater than 0.383 (25%), the predicted risk of recurrence was low. In our study, DWI parameters and their changes after MWA correlated with prognosis. Our study also confirmed the predictive value of tumor location and tumor size for HCC prognosis, but tumor location was excluded from predicting total recurrence, probably since the tumor location was shown to be mainly associated with LTP, and has been included in relatively fewer studies. Nevertheless, tumor size and location are also factors to be considered when using recurrence score to predict LTP or the early appearance of recurrence.

Tumor margin was also shown to be a potential indicator of early recurrence after MWA in our study, and tumors with ill-defined margins are more likely to develop ER after MWA [33]. The rationale behind this observation could be explained by the infiltrative spread of malignant cells into the liver parenchyma and the higher risk of microvascular invasion in ill-defined tumors, which increased the risk of postoperative tumor progression and lowered survival rates [34, 35]. This finding also contributed to the individualized assessment of ablation prognosis due to the difficulty in obtaining peritumoral infiltration by ablation. The present study suggested that a novel image response algorithmic strategy by integrating imaging features showed good predictive performance for the ER. Combined with the tumor size and MAM, the sensitivity and specificity of the recurrence score in predicting ER significantly improved compared with MAM alone.

To evaluate the clinical efficacy of the recurrence score, we performed an exploratory subgroup analysis based on postoperative MAM, tumor size, and tumor differentiation, and the recurrence score was able to give a clearer interpretation of the results. MAM is an important evaluation indicator of ablation efficacy, and when MAM was more than 5 mm, the efficiency of ablation was often considered good based on clinical experience. However, the recurrence score was able to identify about 32% of patients who were still at a high risk of recurrence despite having a good MAM. The prognosis was ambiguous for patients with intermediate differentiation; the patients were often predicted to have a poor outcome, and the recurrence score allowed us to screen approximately 50% of patients who were likely to be in the high-risk recurrence group. Although our study classified intermediate to high differentiation as an intermediate differentiation group, the results were still conclusive. For smaller tumors, the effect of the recurrence score was relatively small, probably because of the high rate of complete ablation.

We are aware of some limitations of our study. First, our retrospective design may have been a source of heterogeneity. Although this is an inherent limitation of all retrospective studies, the ablation procedure and the already standardized nature of MWA procedures over the past 10 years have likely maintained the accuracy of our data. Second, while our study was a single-center, small-sample study, it was an initial exploration of DWI for predicting early recurrent metastasis after ablation, and a prospective multicenter study is needed to validate it. We did not perform histogram analysis, and we used the maximum level method to measure the mean of the quantitative DWI parameters of the lesions. We hypothesize that further studies are needed to assess the added value of histogram analysis. Finally, the degree of pathological differentiation of HCC was not available in a sufficient number of patients to be integrated into such multifactorial analyses.

## Conclusion

In conclusion, our study highlights the ability of an image response algorithmic strategy based on preoperative multiphase enhanced MRI to predict the emergence of ER after MWA of HCC with high sensitivity and specificity. Further multicenter studies with a higher number of patients are needed to validate our findings.

## Supplementary Information

**Additional file 1.**

## Data Availability

The datasets used and analyzed in the current study are available from the corresponding author on reasonable request.

## Abbreviations

HCC: Hepatocellular carcinoma
HR: Hazard ratio
LTP: Local tumor progression
MWA: Microwave ablation
ROC: Receiver operating characteristic
ADC: Apparent diffusion coefficient
EADC: Exponential apparent diffusion coefficient
US-PMWA: Ultrasound-guided percutaneous microwave ablation

## References

[1] Forner A, Reig M, Bruix J (2018). Hepatocellular carcinoma. Lancet.

[2] Choo SP, Tan WL, Goh B, Tai WM, Zhu AX (2016). Comparison of hepatocellular carcinoma in Eastern versus Western populations. Cancer.

[3] Barat M, Fohlen A, Cassinotto C, Jannot AS, Dautry R, Pelage JP (2017). One-month apparent diffusion coefficient correlates with response to radiofrequency ablation of hepatocellular carcinoma. J Magn Reson Imaging.

[4] Nault JC, Sutter O, Nahon P, Ganne-Carrié N, Séror O (2018). Percutaneous treatment of hepatocellular carcinoma: State of the art and innovations. J Hepatol.

[5] Poon RT, Fan ST, Lo CM, Liu CL, Wong J (1999). Intrahepatic recurrence after curative resection of hepatocellular carcinoma: long-term results of treatment and prognostic factors. Ann Surg.

[6] Tabrizian P, Jibara G, Shrager B, Schwartz M, Roayaie S (2015). Recurrence of hepatocellular cancer after resection: patterns, treatments, and prognosis. Ann Surg.

[7] Rimola J, Forner A, Sapena V, Llarch N, Darnell A, Díaz A (2020). Performance of gadoxetic acid MRI and diffusion-weighted imaging for the diagnosis of early recurrence of hepatocellular carcinoma. Eur Radiol.

[8] Kim SW, Joo I, Kim HC, Ahn SJ, Kang HJ, Jeon SK (2020). LI-RADS treatment response categorization on gadoxetic acid-enhanced MRI: diagnostic performance compared to mRECIST and added value of ancillary features. Eur Radiol.

[9] Filippiadis DK, Spiliopoulos S, Konstantos C, Reppas L, Kelekis A, Brountzos E (2018). Computed tomography-guided percutaneous microwave ablation of hepatocellular carcinoma in challenging locations: safety and efficacy of high-power microwave platforms. Int J Hyperthermia.

[10] Kim DH, Choi SH, Kim DW, Lee SS, Lim YS, Kim SY (2021). Combined hepatocellular-cholangiocarcinoma: magnetic resonance imaging features and prognosis according to risk factors for hepatocellular carcinoma. J Magn Reson Imaging.

[11] An C, Park MS, Jeon HM, Kim YE, Chung WS, Chung YE (2012). Prediction of the histopathological grade of hepatocellular carcinoma using qualitative diffusion-weighted, dynamic, and hepatobiliary phase MRI. Eur Radiol.

[12] Jiang T, Xu JH, Zou Y, Chen R, Peng LR, Zhou ZD (2017). Diffusion-weighted imaging (DWI) of hepatocellular carcinomas: a retrospective analysis of the correlation between qualitative and quantitative DWI and tumour grade. Clin Radiol.

[13] Lv X, Chen M, Kong C, Shu G, Meng M, Ye W (2021). Construction of a novel radiomics nomogram for the prediction of aggressive intrasegmental recurrence of HCC after radiofrequency ablation. Eur J Radiol.

[14] Rhee H, Cho ES, Nahm JH, Jang M, Chung YE, Baek SE (2021). Gadoxetic acid-enhanced MRI of macrotrabecular-massive hepatocellular carcinoma and its prognostic implications. J Hepatol.

[15] Ronald J, Wang Q, Choi SS, Suhocki PV, Hall MD, Smith TP (2018). Albumin-bilirubin grade versus MELD score for predicting survival after transjugular intrahepatic portosystemic shunt (TIPS) creation. Diagn Interv Imaging.

[16] Huang Z, Xu X, Meng X, Hou Z, Liu F, Hua Q (2015). Correlations between ADC values and molecular markers of Ki-67 and HIF-1α in hepatocellular carcinoma. Eur J Radiol.

[17] Ahmed M, Solbiati L, Brace CL, Breen DJ, Callstrom MR, Charboneau JW (2014). Image-guided tumor ablation: standardization of terminology and reporting criteria–a 10-year update. Radiology.

[18] Nakai Y, Gonoi W, Kurokawa R, Nishioka Y, Abe H, Arita J (2020). MRI findings of liver parenchyma peripheral to colorectal liver metastasis: a potential predictor of long-term prognosis. Radiology.

[19] Lee HA, Lee YS, Kim BK, Jung YK, Kim SU, Park JY (2021). Change in the recurrence pattern and predictors over time after complete cure of hepatocellular carcinoma. Gut Liver.

[20] Feng RM, Zong YN, Cao SM, Xu RH (2019). Current cancer situation in China: good or bad news from the 2018 Global Cancer Statistics. Cancer Commun (Lond).

[21] Teng W, Liu KW, Lin CC, Jeng WJ, Chen WT, Sheen IS (2015). Insufficient ablative margin determined by early computed tomography may predict the recurrence of hepatocellular carcinoma after radiofrequency ablation. Liver Cancer.

[22] Livraghi T, Meloni F, Di Stasi M, Rolle E, Solbiati L, Tinelli C (2008). Sustained complete response and complications rates after radiofrequency ablation of very early hepatocellular carcinoma in cirrhosis: Is resection still the treatment of choice. Hepatology.

[23] Sun X, Li L, Lyu N, Mu L, Lai J, Zhao M (2020). Follow-up schedule for initial recurrent hepatocellular carcinoma after ablation based on risk classification. Cancer Imaging.

[24] Zhang Z, Zhang Y, Zhang L, Pei Y, Wu Y, Liang H (2019). Incomplete radiofrequency ablation provokes colorectal cancer liver metastases through heat shock response by PKCα/Fra-1 pathway. Cancer Biol Med.

[25] Nakazawa T, Kokubu S, Shibuya A, Ono K, Watanabe M, Hidaka H (2007). Radiofrequency ablation of hepatocellular carcinoma: correlation between local tumor progression after ablation and ablative margin. AJR Am J Roentgenol.

[26] Lee S, Kim SH, Hwang JA, Lee JE, Ha SY (2019). Pre-operative ADC predicts early recurrence of HCC after curative resection. Eur Radiol.

[27] Deckers F, De Foer B, Van Mieghem F, Botelberge T, Weytjens R, Padhani A (2014). Apparent diffusion coefficient measurements as very early predictive markers of response to chemotherapy in hepatic metastasis: a preliminary investigation of reproducibility and diagnostic value. J Magn Reson Imaging.

[28] Gluskin JS, Chegai F, Monti S, Squillaci E, Mannelli L (2016). Hepatocellular carcinoma and diffusion-weighted MRI: detection and evaluation of treatment response. J Cancer.

[29] Li X, Zhang K, Shi Y, Wang F, Meng X (2016). Correlations between the minimum and mean apparent diffusion coefficient values of hepatocellular carcinoma and tumor grade. J Magn Reson Imaging.

[30] Vandecaveye V, Michielsen K, De Keyzer F, Laleman W, Komuta M, Opdebeeck K (2014). Chemoembolization for hepatocellular carcinoma: 1-month response determined with apparent diffusion coefficient is an independent predictor of outcome. Radiology.

[31] Shaghaghi M, Aliyari Ghasabeh M, Ameli S, Ghadimi M, Hazhirkarzar B, Rezvani Habibabadi R (2021). Post-TACE changes in ADC histogram predict overall and transplant-free survival in patients with well-defined HCC: a retrospective cohort with up to 10 years follow-up. Eur Radiol.

[32] Bonekamp S, Jolepalem P, Lazo M, Gulsun MA, Kiraly AP, Kamel IR (2011). Hepatocellular carcinoma: response to TACE assessed with semiautomated volumetric and functional analysis of diffusion-weighted and contrast-enhanced MR imaging data. Radiology.

[33] Shaghaghi M, AliyariG Hasabeh M, Ameli S, Ghadimi M, Hazhirkarzar B, Rezvani Habibabadi R (2020). Role of tumor margin and ADC change in defining the need for additional treatments after the first TACE in patients with unresectable HCC. Eur J Radiol.

[34] Kneuertz PJ, Demirjian A, Firoozmand A, Corona-Villalobos C, Bhagat N, Herman J (2012). Diffuse infiltrative hepatocellular carcinoma: assessment of presentation, treatment, and outcomes. Ann Surg Oncol.

[35] Hu H, Zheng Q, Huang Y, Huang XW, Lai ZC, Liu J (2017). A non-smooth tumor margin on preoperative imaging assesses microvascular invasion of hepatocellular carcinoma: A systematic review and meta-analysis. Sci Rep.

