# Supplementary material for: Multiparametric liver MRI for predicting early recurrence of hepatocellular carcinoma after microwave ablation
Source: Cancer Imaging. 2022 Aug 30;22:42. doi: 10.1186/s40644-022-00471-5 (PMC9429304; doi:10.1186/s40644-022-00471-5)
Supplement: Supplementary file 1 — Additional file 1. [file 40644_2022_471_MOESM1_ESM.docx]

Supplemental Online Content

**Multiparametric Liver MRI for Predicting the outcome of Hepatocellular Carcinoma after Microwave Ablation**

1. Supplementary Method

**MRI Protocol**

All examinations were performed using a 3.0-T whole-body MRI system (GE signa 3.0T HDX TWINSP USA). Patients were fasted with water for 4 h and trained to breathe before the scan. MRI plain scan: axial T2WI, T1WI fat suppression sequence, dual echo T1 iso-inverted phase and coronal and sagittal T2WI, DWI scan using EPI imaging mode, TR: 7500 ms, TE: 57.5 ms, layer thickness: 6 mm, layer spacing: 2 mm, matrix: 128×128, FOV: 420 mm × 420 mm, b-values = 0, 100, and 800 s/mm^2^. ADC was calculated using a single exponential function with b-values of 0 and 800 s/mm^2^. DCE-MR imaging, dynamic enhanced LAVA sequence, TR/TE 3.4/1.5 ms, layer thickness 5 mm, Flip Angle 15. The scanning time was 15 s for each phase, which were montages, arterial phase (30 s), portal phase (60 s), equilibrium phase (100 s) and delayed phase (150 s).

**Ablation Margin Assessment**

One week after MWA, two registered radiologists (Z.Z.H, L.S.S.) measured the MAM on DCE-MRI images. In case of disagreement, the decision was left to a third senior physician. Briefly, the MRI images pre- and post-ablative MWA enhancement were displayed side-by-side using the MRI Analysis System (RadiAnt DICOM Viewer). Anatomical markers around the tumor were selected and the distance from the tumor edge to the markers was measured on the enhanced MRI images. For each marker, the distance before the MWA was subtracted from the distance after the MWA to derive the margin at that site; the minimum value was considered the MAM. MAM were measured for 235 tumors (92.9%; 235 out of 253 tumors), while the remaining 18 tumors could not be accurately measured due to image fusion defects.

**Definition of MRI Features**

1. intratumoral artery: discrete arteries within the tumor in arterial phase images;

(b) ill-defined margin: delayed stage tumor margins, when there are outgrowths around the tumor protruding toward the liver parenchyma;

(c) intra-tumor hemorrhage: defined as a hyperintense area on T1-weighted images, with variable signal intensity on T2-weighted images;

(d) tumor parenchymal necrosis: defined as a central area of high-signal intensity on fat-suppressed turbo spin-echo T2-weighted images (or, if not available or insufficient image quality due to motion artifacts, on half-Fourier acquisition single-shot turbo spin-echo T2-weighted images) without enhancement on postcontrast T1-weighted images;

(e) arterial phase peritumoral enhancement: defined as partial crescentic or polygonal enhancement detectable outside the tumor margin, extensive contact with the tumor border in the arterial phase, and iso-intense signal with the background liver parenchyma in the delayed phase;

(f) lack of capsule enhancement: capsule appearance without a visible enhancement ring;

(g) homogeneity: the internal signal of tumor is uniform without obvious signs of hemorrhage and necrosis.

sTable 1: Baseline Characteristics of the Development and Validation Data Sets

| **Characteristic** | Development (n=226) | Validation (n=113) | *p* Value |
| --- | --- | --- | --- |
| **Age (y)** | 64.3±5.5 | 61.2±8.3 | 0.89 |
| **Gender (Male, %)** | 132 (58.2) | 58 (51.3) |  |
| **Tumor size (cm)** |  |  |  |
| ≤3 cm | 147 (65.0) | 79 (69.9) | 0.62 |
| >3 cm | 79 (35.0) | 34 (30.1) |  |
| **Etiology (%)** |  |  | 0.06 |
| HBV | 203 (89.8) | 100 (88.5) |  |
| HCV | 20 (8.9) | 12 (10.6) |  |
| NAFLD | 3 (1.3) | 1 (0.9) |  |
| **Child-Pugh A/B** |  |  | 0.17 |
| **A** | 145 (64.2) | 61 (54.0) |  |
| **8** | 81 (35.8) | 52 (46.0) |  |
| **ALBI stage (%)** |  |  | 0.31 |
| **I** | 128 (56.5) | 60 (53.1) |  |
| **II** | 98 (43.5) | 53 (46.9) |  |
| **AFP (ng/mL)** |  |  | 0.71 |
| >200 | 131 (58.0) | 59 (52.2) |  |
| <200 | 95 (42.0) | 54 (47.8) |  |
| **Number of Tumors (%)** |  |  | 0.87 |
| 1 | 192 (85) | 100 (88.5) |  |
| >1 | 34 (15) | 13 (11.5) |  |
| **Tumor location (%)** |  |  | 0.32 |
| Close to vessel | 63 (28) | 29 (25.7) |  |
| Close to organ/ subscapsular | 34 (15) | 12 (10.6) |  |
| Close to the bile duct | 9 (4.1) | 3 (2.7) |  |
| No special | 120 (52.9) | 69 (61.0) |  |
| **Minimal ablative margin (%)** |  |  | 0.12 |
| ≤5 mm | 72 (32) | 41 (36.3) |  |
| >5 mm | 154 (68) | 72 (63.7) |  |
| **Histology Differentiation (%)** | 147 | 73 | 0.33 |
| Well differentiated | 56 (38) | 33 (45.2) |  |
| Moderately differentiated | 72 (49) | 35 (47.9) |  |
| Poorly differentiated | 19 (13) | 5 (6.9) |  |
| **Tumor Type (%)** |  |  | 0.54 |
| Primary hepatocellular carcinoma | 61 (27) | 32 (28.3) |  |
| Recurrent hepatocellular carcinoma | 165 (73) | 81 (71.7) |  |

**sTable 2: Correlation between significant qualitative and quantitative MRI variables and Ki-67 expression**

| **MRI variables** | Interreader Agreement | Ki-67 high expression (n=93) | Ki-67 low expression (n=127) | *p* value |
| --- | --- | --- | --- | --- |
| Intratumoral artery | 0.85 | 52 (56) | 41 (32) | 0.01 |
| Ill-defined margin | 0.73 | 70 (67.8) | 15 (12) | <0.001 |
| Intra-tumor hemorrhage | 0.71 | 23 (25) | 24 (19) | 0.32 |
| Tumor parenchymal necrosis | 0.74 | 34 (37) | 41 (32) | 0.50 |
| Arterial phase peritumoral enhancement | 0.75 | 44 (45) | 42 (33) | 0.02 |
| Lack of capsule enhancement | 0.72 | 31 (33) | 83 (65) | 0.01 |
| Homogeneity | 0.72 | 36 (39) | 53 (42) | 0.03 |
| Mean ADC (×10^−3^ mm/s) | 0.71 | 1.06±0.24 | 1.51±0.32 | <0.001 |
| △ADC (×10^−3^ mm/s) | 0.71 | 0.20±0.02 | 0.32±0.05 | 0.01 |
| Mean post-ADC (×10^−3^ mm/s) | 0.72 | 0.86±0.09 | 0.90±0.11 | 0.21 |
| Mean EADC | 0.71 | 0.041±0.005 | 0.072±0.004 | <0.001 |
| △EADC | 0.69 | 0.008±0.001 | 0.015±0.002 | 0.03 |
| Mean post-EADC | 0.71 | 0.301±0.004 | 0.352±0.003 | 0.36 |
| Lesion-to-liver ADC ratio | 0.67 | 0.76 (0.26, 2.80) | 0.89 (0.49, 3.25) | 0.10 |
| Lesion-to-liver EADC ratio | 0.65 | 0.73 (0.25, 2.73) | 0.90 (0.48,3.63) | 0.53 |

Note: unless indicated otherwise, data are the number of patients and data in parentheses are the percentages. a data are mean values ± standard deviation. NA, data are not available. ADC: apparent diffusion coefficient

Note: Data are means ± standard deviations or medians with interquartile ranges in parentheses for continuous variables and numbers of patients with percentages in parentheses for categorical variables.

△ADC: Change of ADC value after ablation

△eADC: Change of eADC value after ablation

**sTable 3: Logistic regression of factors associated with ER**

| **MRI Predictor variables** | Univariate | | Multivariate | |
| --- | --- | --- | --- | --- |
|  | OR 95%CI | *p* Value | OR 95%CI | *p* Value |
| Arterial phase peritumoral enhancement | 2.37 (0.68, 5.98) | 0.15 |  |  |
| Ill-defined Margin | 2.23 (1.33, 5.68) | <0.001 | 2.25 (1.31, 6.12) | <0.001 |
| Tumor parenchymal necrosis | 2.41 (0.22, 4.14) | 0.952 |  |  |
| Lack of capsule enhancement | 2.93 (1.24,2.48) | 0.021 | 3.35 (1.22,7.48) | 0.032 |
| Peri-arterial phase enhancement | 1.52 (0.73,2.55) | 0.240 |  |  |
| Mean ADC (≤1.272×10^−3^ mm/s) | 4.21 (1.07,6.89) | 0.005 | 5.52 (1.22, 9.28) | 0.001 |
| △ADC (≤0.283×10^−3^ mm/s) | 2.73 (1.23, 6.95) | <0.001 | 2.95 (1.56, 7.55) | <0.001 |
| Mean eADC (≤0.316) | 1.32 (1.22, 3.41) | <0.05 | 1.12 (1.25, 6.08) | <0.05 |
| △eADC (≤0.026) | 1.25 (0.65, 2.87) | 0.330 |  |  |

Note: Data are means ± standard deviations or medians with interquartile ranges in parentheses for continuous variables and numbers of patients with percentages in parentheses for categorical variables.
